# Macrophage origin limits functional plasticity in helminth-bacterial co-infection

**DOI:** 10.1371/journal.ppat.1006233

**Published:** 2017-03-23

**Authors:** Dominik Rückerl, Sharon M. Campbell, Sheelagh Duncan, Tara E. Sutherland, Stephen J. Jenkins, James P. Hewitson, Tom A. Barr, Lucy H. Jackson-Jones, Rick M. Maizels, Judith E. Allen

**Affiliations:** 1 Centre for Immunity, Infection and Evolution, School of Biological Sciences, University of Edinburgh, Edinburgh, United Kingdom; 2 Faculty of Biology, Medicine and Health, School of Biological Sciences, University of Manchester, Manchester, United Kingdom; 3 Centre for Inflammation Research, School of Clinical Sciences, University of Edinburgh, Edinburgh, United Kingdom; 4 Centre for Immunology and Infection, University of York, York, United Kingdom; 5 Institute of Infection, Immunity and Inflammation, University of Glasgow, Glasgow, United Kingdom; 6 Centre for Cardiovascular Science, School of Clinical Sciences, University of Edinburgh, Edinburgh, United Kingdom; Max-Planck-Institut of Immunobiology and Epigenetics, GERMANY

## Abstract

Rapid reprogramming of the macrophage activation phenotype is considered important in the defense against consecutive infection with diverse infectious agents. However, in the setting of persistent, chronic infection the functional importance of macrophage-intrinsic adaptation to changing environments vs. recruitment of new macrophages remains unclear. Here we show that resident peritoneal macrophages expanded by infection with the nematode *Heligmosomoides polygyrus bakeri* altered their activation phenotype in response to infection with *Salmonella enterica* ser. Typhimurium in vitro and in vivo. The nematode-expanded resident F4/80^high^ macrophages efficiently upregulated bacterial induced effector molecules (e.g. MHC-II, NOS2) similarly to newly recruited monocyte-derived macrophages. Nonetheless, recruitment of blood monocyte-derived macrophages to *Salmonella* infection occurred with equal magnitude in co-infected animals and caused displacement of the nematode-expanded, tissue resident-derived macrophages from the peritoneal cavity. Global gene expression analysis revealed that although nematode-expanded resident F4/80^high^ macrophages made an anti-bacterial response, this was muted as compared to newly recruited F4/80^low^ macrophages. However, the F4/80^high^ macrophages adopted unique functional characteristics that included enhanced neutrophil-stimulating chemokine production. Thus, our data provide important evidence that plastic adaptation of MΦ activation does occur in vivo, but that cellular plasticity is outweighed by functional capabilities specific to the tissue origin of the cell.

## Author summary

Macrophages are specialized cells of the immune system that help to keep the organism healthy by removing dead cells and debris, assisting in wound healing and fighting off many types of infections as well as tumours. To deal with these various tasks, macrophages produce distinct sets of activation molecules, tailored to the specific task at hand. Once a macrophage displays a certain activation profile it will be less suitable and potentially even detrimental to other typical macrophage tasks. However, previous reports have indicated that macrophages can lose their activation state and quickly switch to a different set of molecules should a new task arise. Here we show that in the context of consecutive co-infection with parasitic worms and bacteria the loss of one set of molecules and gain of another is limited and is determined by whether the macrophage originates from the affected tissue or has been newly recruited to the site of infection. Taken together our data indicates that tissue origin of a macrophage has a dominant impact on macrophage activation and treatments aiming to alter macrophage activation status (e.g. in cancer) need to consider the source of the cells in order to successfully predict outcome.

## Introduction

Macrophages (MΦ) are central to many immune and homeostatic processes and adopt a variety of activation phenotypes. During bacterial infections ‘classically activated’ MΦ produce anti-microbial effector molecules, exhibit enhanced antigen presentation capacity and produce proinflammatory cytokines. In contrast, during helminth infection MΦ are activated by IL-4Rα-signaling and called M(IL-4), or ‘alternatively activated’ [1,2]. M(IL-4) express low levels of co-stimulatory molecules, produce molecules associated with wound healing, and are considered anti-inflammatory [1]. Of note, the MΦ activation phenotype is not fixed and the prevailing view is that MΦ can adopt a variety of activation states in response to their environment [3–6].

Although MΦ plasticity is well established, most of the data supporting this concept are based on in vitro findings or *ex vivo* derived cells. Moreover, full activation, especially in infectious settings in vivo, rarely occurs in 100% of the MΦ present [7–9]. Thus, the observed plasticity might be due to hitherto quiescent subsets of MΦ responding rather than true plasticity of previously activated cells. Furthermore, recent data have highlighted distinct mechanisms for MΦ accumulation in different infection settings [10]. During bacterial infection bone marrow-derived blood monocytes infiltrate from the vasculature and differentiate into anti-bacterial effector MΦ or dendritic cells (DC) [11,12] while the tissue resident MΦ population is usually lost from the site of infection in a process called the MΦ disappearance reaction [11,13,14]. In contrast the type 2 immune response associated with helminth infection can result in proliferative expansion of tissue resident MΦ with minimal recruitment of monocyte-derived cells [8,15]. Of note tissue resident peritoneal MΦ can originate from either prenatal sources or bone marrow derived precursors depending on the age of the animal [16,17] but the use of the term ‘origin’ in the context of this study refers to the tissue of origin (blood vs. peritoneal cavity) within the time frame of the infection. Critically, the functional significance of resident cell expansion vs monocyte recruitment is not yet clear. The finding that some helminth infections also lead to recruitment of blood monocytes [9] and that monocyte-recruited MΦ show marked disparity in the transcriptional response to recombinant interleukin 4 (IL-4) as compared to tissue resident MΦ [18], suggests important functional differences. Thus, the distinct tissue origin of MΦ during infection combined with a current lack of in vivo evidence for cell-intrinsic changes in activation state raised two questions. 1) Does MΦ activation plasticity occur in vivo at the level of the individual cell and 2) is plasticity and/or MΦ origin relevant to infection outcome?

To address these questions, we turned to co-infection models, which provide physiologically relevant insight into MΦ polarization and plasticity [19]. We utilized murine co-infection with *Heligmosomoides polygyrus bakeri* (*H*. *polygyrus*) and attenuated *Salmonella enterica* subsp. *enterica* serovar Typhimurium (*S*. *enterica* ser. Typhimurium; SL3261, ΔaroA). *H*. *polygyrus* is a natural gastrointestinal nematode parasite of mice inducing a strong type 2 immune response; infection leads to pronounced proliferative expansion and M(IL-4) activation of tissue resident MΦ in the peritoneal cavity [10,15,20]. Infection with Salmonella species induces a potent pro-inflammatory response required for bacterial clearance [21]. In co-infection experiments, mice were infected with *H*. *polygyrus* and at peak expansion of resident peritoneal MΦ SL3261 was inoculated i.p. Cell-intrinsic plasticity of the M(IL-4) MΦ was observed in response to bacterial infection but plasticity did not appear relevant for protection within the first 5 days of SL3261 infection. Indeed, inflammatory recruitment of monocyte-derived MΦ and neutrophils was unhampered in co-infected mice. Moreover, the inflammatory influx was accompanied by the marked disappearance of the helminth-expanded resident MΦ. Microarray and functional analyses demonstrated that nematode-expanded MΦ do adapt their activation phenotype in co-infection settings, but the tissue origin places limitations on the functional response of these cells.

## Results

### Nematode-elicited MΦ can be classically activated independent of prior activation state

**Fig 1:**
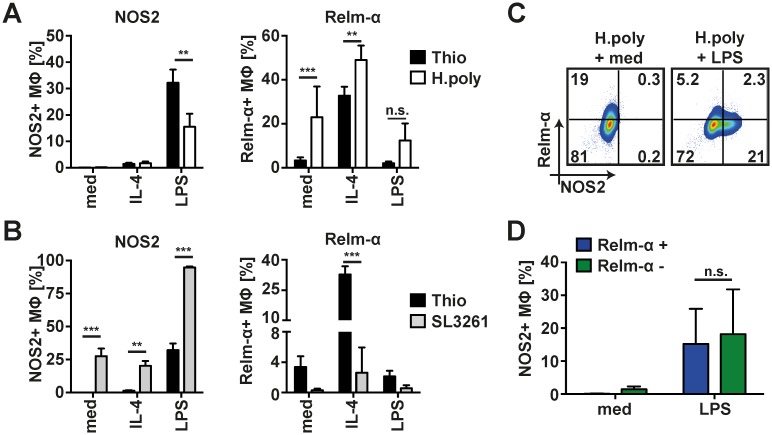
*H*. *polygyrus* derived peritoneal MΦ can be re-polarized to express NOS2 in vitro. (A) In vitro stimulation of whole PEC isolated 14 days after *H*. *polygyrus* infection and stimulated with rIL-4 (IL-4), LPS (LPS) or without additional stimulus (med) for 24h. Thioglycollate-elicited MΦ were used as control. Expression of intracellular NOS2 and Relm-α in CD115+CD11b+ MΦ measured by flowcytometry. (B) As A using whole PEC isolated from *S*. *enterica* ser. Typhimurium (SL3261) infected animals 14 days after infection. (C) Representative dot plots of NOS2 expression versus Relm-α expression in MΦ (CD11b+CD115+) isolated in A. (D) Quantification of the data depicted in C representing NOS2 expression in Relm-α positive (blue) or negative (green) MΦ from *H*. *polygyrus* infected animals. Bars depict mean and SEM of 4–5 animals per group. One representative experiment of three depicted. **: p<0.01, ***; p<0.001; n.s.: not significant.

In order to examine the capacity of nematode-elicited MΦ (NeMΦ) to change their M(IL-4) phenotype, peritoneal exudate cells from mice infected 14 days previously with *H*. *polygyrus* were stimulated in vitro with lipopolysaccharide (LPS), recombinant IL-4 (rIL-4) or without additional stimulus. As a reference for the magnitude of the response we used non-polarised thioglycollate-elicited peritoneal MΦ, which respond well to polarising stimuli [22]. LPS stimulation induced upregulation of NOS2 expression in NeMΦ (Fig 1A), which varied in magnitude relative to control thioglycollate-elicited MΦ between experiments (S1 Fig). Importantly, induction of NOS2 expression in NeMΦ was consistently observed, indicating that NeMΦ retain their capacity to respond to bacterial stimuli. NeMΦ also remained responsive to rIL-4 as demonstrated by enhanced Relm-α expression (Fig 1A). Of note, re-polarization of classically activated MΦ was less evident; MΦ isolated from animals harbouring a bacterial infection (*S*. *enterica* ser. Typhimurium, SL3261) showed strong upregulation of NOS2 expression following stimulation with LPS, notably in excess of the response observed in thioglycollate elicited MΦ (Fig 1B). In contrast only very limited non-significant upregulation of Relm-α was observed in response to rIL-4 (Fig 1B). Thus, bacterial stimuli seemed to provide a more restrictive activation signal, placing limitations on the plasticity of the response and favouring anti-bacterial outcomes of activation.

To assess whether the upregulation of NOS2 in NeMΦ was restricted to a subpopulation that had not previously responded to IL-4Rα signaling, we analysed co-expression of NOS2 and Relm-α (Fig 1C). Irrespective of whether the isolated MΦ expressed Relm-α or not both populations showed equal upregulation of NOS2 in response to LPS (Fig 1D) indicating that classical activation was not restricted to previously non-responding cells.

To directly confirm that M(IL-4) can switch their phenotype in vivo, resident MΦ that had been expanded and activated by in vivo delivery of IL-4 complex, were transferred into the peritoneal cavity of SL3261 infected animals. The transferred cells showed equivalent induction of NOS2 expression as host MΦ (S1 Fig). Thus, activation plasticity of M(IL-4) also occurred in vivo despite potential competition with host MΦ for activating stimuli

### Bacterial co-infection leads to the loss of F4/80^high^ nematode-expanded MΦ

As described previously *H*. *polygyrus* infection leads to the proliferative expansion of peritoneal, tissue resident MΦ [15] whereas *S*. *enterica* ser. Typhimurium induces influx of blood monocyte-derived MΦ [23]. Thus, to address whether the presence of large numbers of nematode expanded, tissue resident MΦ, in an M(IL-4) activation state, influenced bacterial induced recruitment of cells (gating strategy depicted in (S2 Fig) we established a consecutive co-infection model. Mice were orally infected with *H*. *polygyrus* followed 9 days later by i.p. injection of SL3261 (S3A Fig). In this co-infection model peak expansion and accumulation of *H*. *polygyrus* driven M(IL-4) occurs before bacterial challenge (S3B Fig). Effects on MΦ were analysed 5 days later, a timepoint when T cell-independent, partially NOS2-dependent control of bacterial growth can be observed [24].

**Fig 2:**
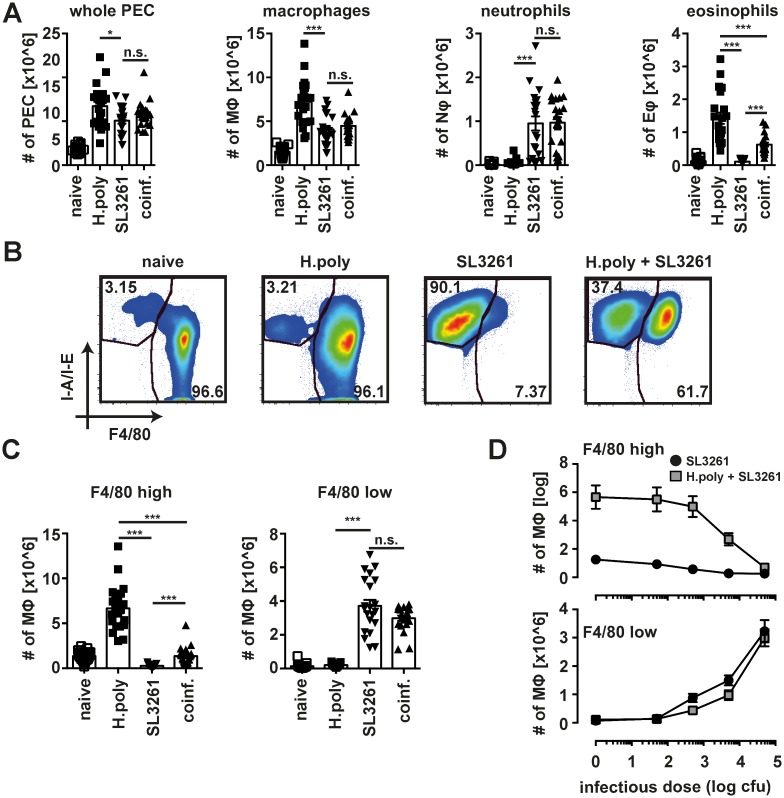
Bacterial co-infection triggers the disappearance reaction in helminth expanded MΦ. (A) Number of total peritoneal exudate cells, MΦ, eosinophils and neutrophils in uninfected (naïve) individually (SL3261 and H.poly) or co-infected mice (B) Representative dot plots depicting MΦ isolated in A. (C) Number of F4/80^high^ and F4/80^low^ MΦ from mice analysed in A. Data pooled from four separate experiments using 5–6 animals per group. Datapoints depict individual animals and bars indicate mean and SEM. (D) Number of peritoneal F4/80^high^ and F4/80^low^ MΦ in single (black circles, SL3261) or co-infected mice (grey squares, H.poly + SL3261) with the indicated doses of SL3261. Animals not receiving bacterial inocula (naïve and single *H*. *polygyrus* infection) are included with an arbitrary infection dose of 1 CFU SL3261 to allow depiction on a log scale. Data pooled from two separate experiments with 5 animals per group each. *: p<0.05; **: p<0.01, ***; p<0.001; n.s.: not significant.

All infections significantly increased the total number of cells in the peritoneal cavity as compared to naïve animals (Fig 2A). As expected there was preferential influx of eosinophils in nematode infected animals as compared to a more neutrophilic inflammation in SL3261 infected mice. In co-infected animals neutrophil influx was not impeded by prior *H*. *polygyrus* infection indicating normal recruitment of these cells from the circulation. In contrast the number of eosinophils was reduced by the presence of bacteria confirming crossregulation between the anti-helminth and anti-bacterial immune response in our model. The number of MΦ in the peritoneal cavity increased both in bacterial and helminth infection, but co-infection had no additive effect (Fig 2A). Consistent with the differences in tissue origin, MΦ accumulating in singly-infected nematode or bacterial infections differed phenotypically [8], expressing high or low surface levels of F4/80 respectively (Fig 2B). Consecutive co-infection led to the simultaneous appearance of both MΦ populations (F4/80^high^ and F4/80^low^) (Fig 2C). Furthermore, similar to neutrophils, F4/80^low^ MΦ were expanded to an equivalent level between single SL3261 and co-infected animals (Fig 2C). At the same time a significant reduction in the number of F4/80^high^ MΦ was observed in co-infected relative to single *H*. *polygyrus* infected animals. Moreover, the degree of F4/80^low^ cell recruitment depended on the inoculating dose of SL3261 and correlated with the loss of F4/80^high^ MΦ (Fig 2D).

### Nematode-expanded MΦ do not alter resistance to SL3261 infection and efficiently upregulate anti-bacterial effector markers in response to bacterial infection

**Fig 3:**
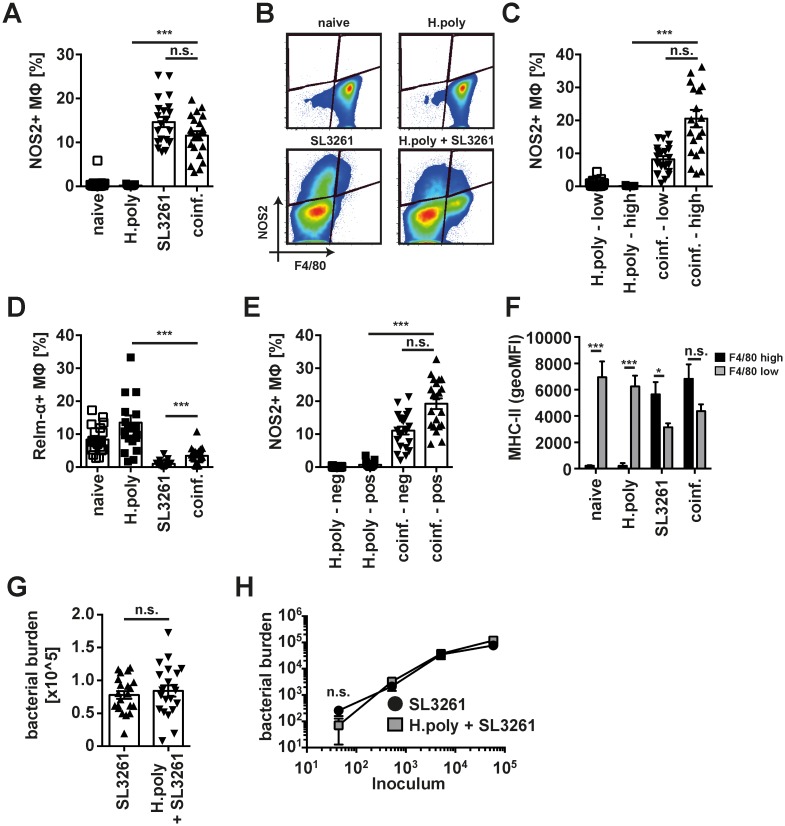
Helminth-expanded MΦ upregulate NOS2 and MHC-II expression following bacterial infection. (A) Intracellular NOS2 staining in peritoneal MΦ from SL3261 (SL3261), *H*. *polygyrus* (H.poly) co-infected (coinf.) or naive mice. (B) Representative dot plots depicting NOS2 vs. F4/80 expression in peritoneal MΦ isolated in A. (C) Quantitative analysis of NOS2 expression in F4/80^high^ (high) or F4/80^low^ (low) MΦ isolated in A (D) Intracellular Relm-α staining in MΦ isolated in A. (E) Quantitative analysis of NOS2 expression in Relm-α positive (pos) or negative (neg) MΦ isolated in A (F) MHC-II expression (geometric mean fluorescence intensity) on F4/80^high^ and F4/80^low^ MΦ isolated in A. (G) Splenic bacterial burden in single SL3261 (SL3261) or consecutively *H*. *polygyrus*.*/* SL3261 co-infected (H.poly + SL3261) mice. Data pooled from 4 separate experiments with 5–6 animals per group per experiment. Datapoints depict individual animals and bars indicate mean and SEM. (H) As in G utilizing indicated infection doses of SL3261. One representative experiment of 2 with 5 animals per group shown. *: p<0.05; **: p<0.01, ***; p<0.001; n.s.: not significant.

The loss of F4/80^high^ MΦ and concomitant recruitment of F4/80^low^ MΦ made us question whether the plastic response of NeMΦ observed in vitro (Fig 1) did occur in vivo. Utilising the consecutive co-infection model described above we found no statistical difference in the induction of intracellular NOS2 expression by peritoneal MΦ in single SL3261 or co-infected animals (Fig 3A). Furthermore, induction of NOS2 was not restricted to newly recruited F4/80^low^ MΦ, as F4/80^high^ MΦ showed at least equal if not enhanced capacity to induce NOS2 expression (Fig 3B & 3C). Moreover, SL3261 co-infection led to loss of the M(IL-4) activation phenotype in NeMΦ, as measured by intracellular Relm-α expression, but induction of NOS2 was independent of previous M(IL-4) activation as indicated by equivalent expression in Relm-α positive and negative cells (Fig 3D & 3E). Thus, nematode expanded F4/80^high^, resident-derived MΦ in co-infected animals showed clear and efficient induction of anti-bacterial effector mechanisms, which was further evidenced by enhanced expression of MHC-II (Fig 3F). In line with the upregulation of anti-bacterial effector molecules by F4/80^high^ MΦ and unaltered recruitment of F4/80^low^ MΦ, no significant difference was found in the number of bacteria present in the spleen of co-infected animals as compared to single SL3261 infected animals (Fig 3G). Thus, the more than 7-fold greater number of MΦ present in the peritoneal cavity of *H*. *polygyrus* infected mice at the time of bacterial inoculation (S3B Fig) did not provide any protection, despite their apparent activation plasticity. We considered that the inability to provide protection may be due to injection of relatively large quantities of bacteria. We therefore titrated the dose of SL3261 but no effect of helminth infection on splenic bacterial burden was detected, even when only 50 bacteria were injected into the peritoneal cavity (Fig 3H). Similarly, when mice were co-infected with high doses of SL3261 (~3x10^6 CFU), no difference in resistance was observed (S4 Fig).

### F4/80^low^ cells do not arise from plastic conversion of F4/80^high^ MΦ

**Fig 4:**
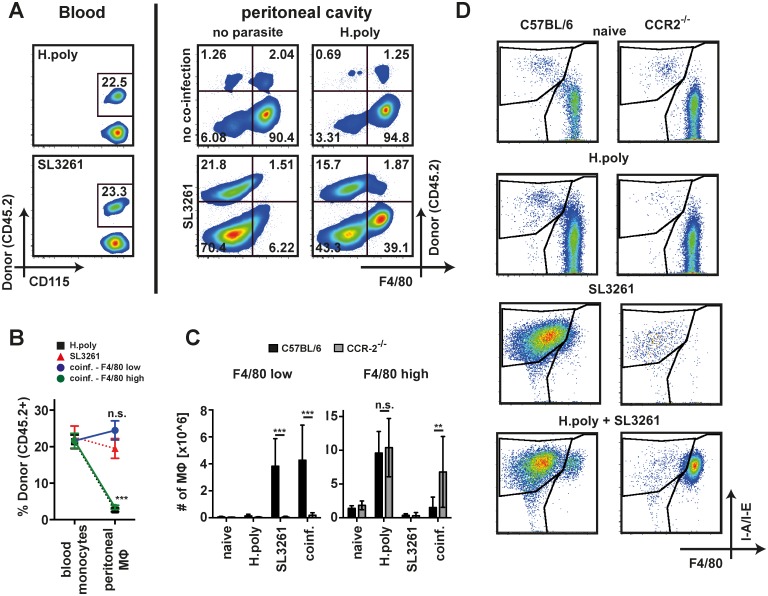
Bacterial co-infection leads to recruitment of MΦ from the vasculature. (A) Representative flow diagrams depicting CD45.2 chimerism in Ly6C^high^ blood monocytes (CD11b+ CD115+ Ly6C^high^) or peritoneal MΦ (lineage- CD11c- F4/80+MHC+) in Cd45.1 host mice after partially shielded γ-irradiation, and subsequent single (top right, bottom left) or co-infection (bottom right). (B) Quantitative analysis of chimerism in Ly6C^high^ blood monocytes and F4/80^high^ (green circles) or F4/80^low^ (blue circles) MΦ in co-infected mice analysed in E. As comparison chimerism of total MΦ in single *H*. *polygyrus* (filled squares) or SL3261 (red triangles) infected animals is shown. Data pooled from two separate experiments with 5 animals per group each. Asterisks indicate statistical differences between blood monocytes and peritoneal MΦ in co-infected animals. (C) Number of F4/80^high^ and F4/80^low^ peritoneal MΦ in SL3261 (SL3261), *H*. *polygyrus* (H.poly) co-infected (coinf.) or naive C57BL/6 (black bars) or *Ccr2*-/- mice (grey bars). Data pooled from two independent experiments with 4–6 mice per group. (D) Representative dot plots depicting MΦ isolated in C. **: p<0.01, ***; p<0.001; n.s.: not significant.

F4/80^low^, blood monocyte derived MΦ exposed to tissue specific factors can give rise to tissue resident F4/80^high^ MΦ [16,17]. Thus, whilst the loss of F4/80^high^ MΦ may be due to their disappearance or death, a conversion of F4/80^high^ nematode-expanded MΦ to F4/80^low^ MΦ could have occurred under co-infection settings. To discriminate between these possibilities we utilized partially protected chimeras in which the peritoneal cavity is shielded from lethal irradiation [8,25]. This protects the tissue resident peritoneal population and prevents their replacement by bone marrow-derived cells but results in a chimeric blood monocyte population (S5 Fig). By comparing the proportion of donor cells within a given MΦ population with the proportion in blood monocytes it can be determined whether the population is derived from the bone marrow (ratio equal to monocytes) or from tissue resident MΦ (low donor ratio). Peritoneal MΦ from single *H*. *polygyrus* or SL3261 infected animals showed low or high proportions of donor cells, respectively, confirming their tissue resident MΦ or bone marrow derived origins (Fig 4A). Importantly the two MΦ populations present in co-infected mice displayed identical chimerism to their respective counterparts in single infected mice. The F4/80^high^ MΦ included a very low percentage of donor bone marrow cells while F4/80^low^ MΦ displayed similar chimerism to blood monocytes (Fig 4A & 4B). The data demonstrate that no conversion between these two MΦ populations occurred and recruitment of anti-bacterial F4/80^low^ MΦ was unperturbed by the presence of large numbers of helminth-expanded F4/80^high^ MΦ.

We further confirmed the blood monocyte origin of F4/80^low^ cells using CCR-2 deficient mice, which fail to recruit MΦ to inflammatory sites in part due to defective egress of monocytes from the bone marrow [26]. Recruitment of F4/80^low^ MΦ in response to SL3261 was ablated in *Ccr2*-/- animals both in single and co-infection whereas expansion of resident F4/80^high^ MΦ was unaltered during *H*. *polygyrus* infection (Fig 4C & 4D). Of note the disappearance of F4/80^high^ cells observed in wildtype mice was much less pronounced in co-infected *Ccr2*-/- animals indicating a link between recruitment of F4/80^low^ MΦ and the disappearance reaction. Enhanced cell death is likely part of the explanation for the loss of F4/80^high^ MΦ as indicated by increased Annexin V staining specifically in this population following SL3261 single or co-infection in wild-type mice (S6 Fig). Thus, F4/80^high^ nematode-expanded MΦ did not convert to an F4/80^low^ phenotype but were displaced from the peritoneal cavity during bacterial co-infection by new, blood monocyte derived MΦ. Notably, the magnitude of blood cell recruitment was the same in single bacterial or co-infected animals.

### SL3261 infection induces a unique gene expression profile in F4/80^high^ MΦ

**Fig 5:**
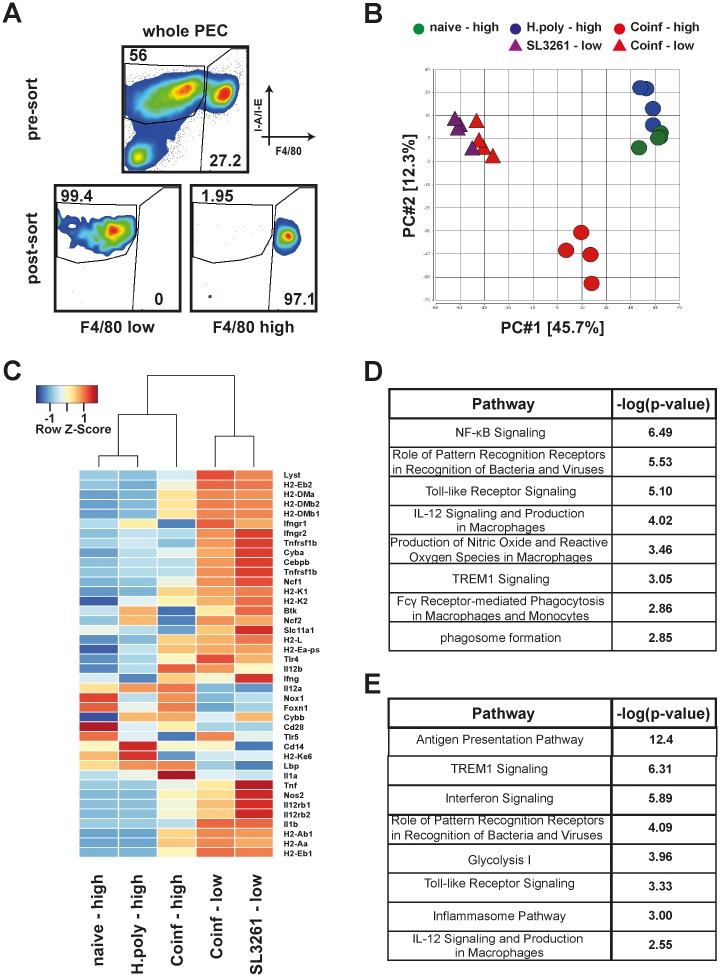
Microarray analysis of MΦ populations from single and co-infected animals. A) Representative dotplots depicting unsorted and sorted MΦ populations. Tissue resident derived, F4/80^high^ and monocyte-derived F4/80^low^ MΦ were identified as lineage negative (Ly6G-, TCRβ-, SiglecF-, CD19-) CD115+ F4/80 high or low, respectively, and sorted by fluorescence activated cell sorting reaching purities of >96%. B) Principal component analysis of global gene expression in F4/80^high^ (circle) or F4/80^low^ (triangle) MΦ from naive (green), single H. polygyrus (blue), SL3261 (purple) or co-infected (red) animals. C) Hierarchical clustering and heatmap of genes associated with resistance to SL3261. D) Selected canonical pathways determined by IPA, significantly enriched for DE genes in pairwise comparisons of F4/80^low^ and F4/80^high^ MΦ from co-infected animals. E) As D) in pairwise comparisons of F4/80^high^ MΦ from co-infected animals compared to single *H*. *polygyrus* infected animals.

The recruitment of monocyte derived MΦ in conjunction with the displacement of F4/80^high^ MΦ following bacterial inoculation of *H*. *polygyrus* infected animals strongly suggested that these two cell populations have distinct capacities and functions during co-infection. To elucidate these differences we subjected F4/80^high^ and F4/80^low^ MΦ populations from co-infected animals to microarray gene expression analysis. Both populations were isolated from the same animal by fluorescence activated cell sorting (Fig 5A). F4/80^high^ MΦ from naïve or *H*. *polygyrus* single infected as well as F4/80^low^ MΦ from SL3261 single infected animals were used as controls. The quality and reproducibility of the data was confirmed by hierarchical clustering analysis of global expression profiles, which grouped according to biological conditions (S7 Fig). Principal component analysis showed F4/80^high^ MΦ in naïve and *H*. *polygyrus* infected animals clustering together, while F4/80^low^ MΦ in single SL3261 or co-infected animals clustered together, reaffirming the different tissue origins of these cell populations. Notably, F4/80^high^ MΦ in co-infected animals clustered separately from either of these populations revealing a unique response profile in response to bacterial infection (Fig 5B).

To specifically address whether F4/80^high^ MΦ could effectively contribute to resistance against SL3261 infection we compared expression of known resistance-associated genes [27] across all experimental groups (Fig 5C). F4/80^low^ MΦ from both single SL3261 and co-infected animals showed similar, largely enhanced expression of these genes. F4/80^high^ MΦ in co-infected animals also showed enhanced expression of most resistance genes, including *Nos2*, as compared to MΦ from naïve and *H*. *polygyrus* infected animals, confirming plastic adaptation of the activation phenotype. However, the overall response in F4/80^high^ MΦ was muted and most resistance-associated genes had less differential expression than in F4/80^low^ MΦ. Specifically *Slc11a1* (i.e. *Nramp*), which was significantly upregulated in F4/80^low^ MΦ by bacterial infection, was virtually unchanged in tissue resident derived MΦ. Co-infection also induced a divergence with regard to the production of IL-1 in which *Il1b* expression was highly increased in F4/80^low^ MΦ while *Il1a* expression was dramatically increased in F4/80^high^ MΦ.

To further highlight functional differences, we performed pathway analyses on differentially expressed (DE) genes (q-value (FDR)<0.01, log2FC ±0.5) in a pairwise comparison of F4/80^high^ and F4/80^low^ MΦ from co-infected animals utilising Ingenuity Pathway Analysis (IPA). To avoid bias caused by different cellular origins, we compared the list of DE genes between F4/80^low^ and F4/80^high^ MΦ in our dataset with DE genes in F4/80^low^ and F4/80^high^ MΦ in naïve animals obtained from publicly available datasets (Immunological Genome Project, http://www.immgen.org [28]) and restricted the analysis to genes unique to the co-infection setting (S8 Fig & S1 Table). This analysis revealed enrichment of DE genes in several pathways associated with anti-bacterial or pro-inflammatory responses (Fig 5D). The pattern of gene expression between F4/80^low^ and F4/80^high^ MΦ within these pathways provided further evidence that the anti-bacterial response was less potent in F4/80^high^ MΦ during co-infection. Of note, pairwise comparison of F4/80^high^ MΦ from co-infected vs F4/80^high^ MΦ from single *H*. *polygyrus* infected animals revealed anti-microbial pathways as differentially up-regulated (Fig 5E). Once again, this confirmed that F4/80^high^ MΦ during co-infection adopt anti-microbial characteristics. Interestingly, other affected pathways in F4/80^high^ MΦ from co-infected vs. single *H*. *polygyrus* infected animals included several pathways associated with induction of apoptosis (S9 Fig). Alongside the pronounced Annexin V staining (S6 Fig) these data suggest enhanced cell death as a major contributor to the MΦ disappearance reaction.

Overall the gene expression analysis revealed that F4/80^high^, parasite expanded MΦ altered their activation phenotype to adapt to the bacterial infection. However, the anti-bacterial response was limited in comparison to monocyte-derived cells.

### *H*. *polygyrus* expanded F4/80^high^ MΦ retain tissue-sentinel / neutrophil recruiting properties

**Fig 6:**
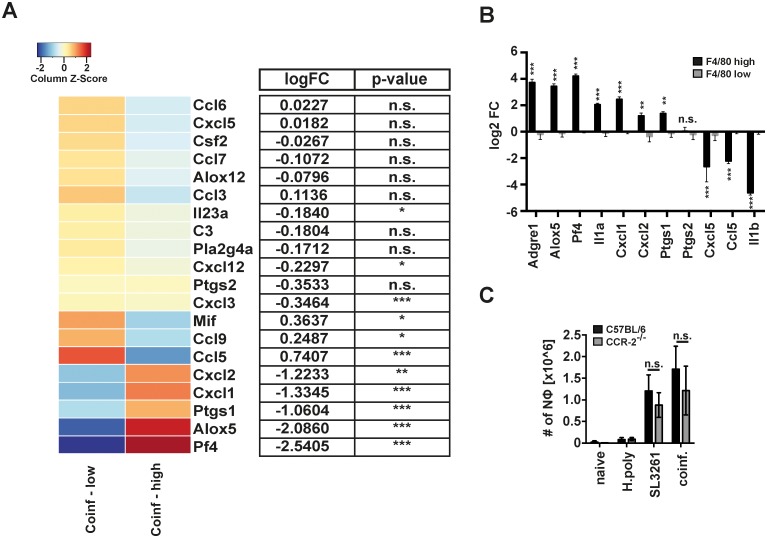
F4/80^high^ MΦ preferentially produce neutrophil recruiting factors. (A) Heatmap of relative expression of neutrophil chemotactic factors and enzymes involved in the generation of neutrophil chemotactic factors in F4/80^high^ and F4/80^low^ MΦ from co-infected animals. (B) qRT-PCR of selected chemotactic factors identified in A. One representative experiment of 2 with 6 animals per group shown (C) Number of peritoneal neutrophils in uninfected (naïve) individually (SL3261 and H.poly) or co-infected (coinf.) WT (black bars) or *Ccr2*-/- (grey bars) mice. One representative experiment of 2 shown. **: p<0.01, ***: p<0.001; n.s.: not significant.

Although F4/80^high^ MΦ showed an overall limited anti-bacterial response, certain genes did not follow this pattern (Fig 5C). Specifically *Il1a* showed divergent, unexpectedly enhanced expression (Fig 5C) in F4/80^high^ MΦ from co-infected animals. This made us question whether these cells adopted functionalities other than anti-bacterial effector mechanisms. In this context Schiwon *et al*. recently highlighted a two-step model of inflammation with tissue resident Ly6C- and newly recruited Ly6C+ MΦ adopting different, non-redundant roles in the recruitment and activation of neutrophils during uropathogenic *E*. *coli* infection [29]. In line with these findings F4/80^high^ MΦ from co-infected animals in our model expressed enhanced levels of key neutrophil chemotactic factors (e.g. *Cxcl1*, *Cxcl2*, *Pf4*) [30] or enzymes involved in the generation of neutrophil chemotactic factors (e.g. *Alox5*, *Ptgs1*) (Fig 6A & 6B). Thus, F4/80^high^, helminth expanded tissue resident derived MΦ seemed to retain their tissue sentinel capacity and may contribute to resistance to bacterial infection through recruitment of neutrophils and other inflammatory cells. The distinct capacity of F4/80^high^ resident derived MΦ to promote neutrophil recruitment was supported by data from *Ccr2*-/- mice. Recruitment of neutrophils was only marginally reduced in SL3261 infected or co-infected *Ccr2*-/- mice despite failing to recruit F4/80^low^ MΦ (Figs 6C & 4C).

## Discussion

Inflammatory MΦ are commonly recruited through influx and differentiation of blood monocytes [12,31] whereas some helminth infections, and Th2 cytokines, lead to the proliferative expansion of tissue resident MΦ [10]. The functional importance of this divergence in recruitment is not completely clear, yet helminth infections can last years and avoidance of chronic inflammation might be the evolutionary driver of this phenomenon [10,32]. Furthermore MΦ are well known to flexibly adapt their activation phenotype to changes in their environment in vitro [33,34]. Nonetheless, limited data exist on the true plasticity of individual cells in vivo, and on the relative importance of plasticity vs. cellular recruitment.

Here we show that *H*. *polygyrus—*expanded peritoneal MΦ can effectively upregulate anti-bacterial defense mechanisms (i.e. NOS2, MHC-II) in response to bacterial stimulation in vitro and in vivo. Importantly, plasticity was not restricted to newly recruited or previously non-activated cells. However, the magnitude of the response was limited in the resident population and MΦ instead adopted specific characteristics dependent on their tissue origins. Thus, plasticity of MΦ activation as defined by a change in activation phenotype did exist in vivo, albeit with certain restrictions on the degree of repolarization. Limitations on MΦ activation make evolutionary sense to allow fine tuning of the ensuing immune response depending on the persistence and virulence of the invading pathogen [29].

Altered, often reduced responses of MΦ to a later challenge following primary stimulation have been described before [35]. IL-4 and IFNγ have been shown to induce distinct non-overlapping enhancers of gene transcription which are retained even when stimulation ceases [36]. Hence stimulation of MΦ can generate an epigenetic memory, which influences and dictates future responses [37,38]. Indeed, helminth infections are in general assumed to impart a detrimental effect on resistance to bacterial infections in part by altering MΦ activation [19,39–42]. This is also evident in our findings. F4/80^low^ MΦ from co-infected animals showed reduced induction of anti-bacterial effector genes as compared to F4/80^low^ MΦ from single SL3261 infected animals. However, the limiting effect of previous cytokine exposure was overlaid by the much more profound effect of the immediate origin of the MΦ (tissue resident vs. blood monocyte derived). F4/80^high^ and F4/80^low^ MΦ in co-infected animals responded in unique ways to bacterial infection indicating differing functional roles. Notably, tissue resident colon MΦ, although originally blood monocyte derived, have been shown to maintain a similar restricted and preferentially anti-inflammatory phenotype in the face of inflammation [43] as do the peritoneal MΦ discussed here. Furthermore exposure of MΦ to tissue environmental factors has been described to affect and shape MΦ responses to infection [16]. Therefore, independent of embryonic or bone marrow derived origins, tissue MΦ responses seem largely determined by previous exposure to tissue factors and adoption of a resident phenotype. Thus, plasticity of MΦ activation as defined by the adoption of a full anti-bacterial phenotype by helminth expanded MΦ did not happen in vivo. Rather tissue resident derived MΦ, whether expanded by helminth infection or not, responded in a unique, non-redundant fashion to the bacterial infection likely necessary for an optimal induction of the immune response.

Whether these unique properties of tissue resident MΦ are of functional relevance to the expansion of these cells in some helminth infections [10] but not others [9] remains unclear. Previous studies suggest that early innate recognition of bacteria has the potential to overcome the normally detrimental impact of helminth infection on resistance to bacterial infection [44,45]. Furthermore helminth expanded resident MΦ have been shown to exert a protective effect in models of sepsis [46,47]. Thus, expansion of these cells might serve the dual purpose of rapid initiation of anti-bacterial effector mechanisms while avoiding excessive, potentially self-harming immune activation dependent on the virulence and persistence of the invading pathogen.

In this context it is interesting to note that in our co-infection experiments the disappearance reaction of tissue resident MΦ seemed linked to the recruitment of monocytes and monocyte derived MΦ. Although the exact mechanism remains unclear, recruited MΦ appear to displace the resident population in a time and dose dependent manner likely through the induction of apoptosis. In the light of their activation plasticity and non-redundant role in triggering an anti-bacterial immune response their displacement raises the question of why tissue resident MΦ are removed in a persisting bacterial infection? In line with the muted pro-inflammatory response found here the MΦ disappearance reaction might reflect differences of tissue resident and recruited MΦ in the capacity to deal with intracellular pathogens [48,49] or their interaction with the adaptive immune system [50]. Thus, although relevant in early phases of the immune response, persistence of tissue resident MΦ may render the host more susceptible to the infection or dampen the ensuing T cell response.

Taken together our data indicate that plastic adaptation of MΦ responses to consecutive co-infections does occur in vivo but is outweighed by cellular recruitment due to functional restrictions imposed by the tissue origin of the MΦ.

## Materials and methods

### Ethics statement

All animal experiments were performed in accordance with the UK Animals (Scientific Procedures) Act of 1986 under a Project License (60/4104) granted by the UK Home Office and approved by the University of Edinburgh Ethical Review Committee. Euthanasia was performed by giving an overdose of anaesthetic (Ketamine/Medetomidine; 1/1; v/v).

### Mice and infection

C57BL/6 mice were bred and maintained in specific pathogen–free facilities at the University of Edinburgh. Experimental mice were age and sex matched.

*Heligmosomoides polygyrus bakeri* life cycle was maintained in house and infective third-stage larvae (L3) were obtained as described elsewhere [51]. Mice were infected with 200 *H*. *polygyrus* L3 by oral gavage. Fecal egg burden was determined on day 13 of the infection using a McMaster counting chamber (Hawksley).

The attenuated, aroA deficient *Salmonella enterica* serovar Typhimurium strain SL3261 [52] was cultured as stationary overnight culture from frozen stock in Luria-Bertani broth. Unless indicated otherwise animals were injected i.p. with ~3-5x10^4 CFU diluted in PBS. Infectious doses and splenic bacterial burdens were enumerated by plating inocula or tissue homogenates in 10-fold serial dilutions in PBS on LB-Agar plates.

IL-4–anti–IL-4 mAb complex (IL-4c) was prepared as described previously [53], and mice were injected i.p. with 5 μg of recombinant IL-4 (13.5 kD; PeproTech) complexed to 25 μg 11B11 (Bio X Cell) or 100 μL PBS vehicle control on days 0 and 2, and peritoneal exudate cells were harvested on day 4.

For in vitro experiments mice were injected with 400 μL 4% Brewer modified thioglycollate medium (BD Biosciences) three days prior to necropsy.

### Cell-isolation

Mice were sacrificed by exsanguination via the brachial artery under terminal anesthesia. After sacrifice, peritoneal cavity exudate cells (PEC) were obtained by washing the cavity with 9 mL lavage media comprised of RPMI 1640 containing 1% normal mouse serum (AbD serotec), 100 U/mL penicillin and 100 μg/mL streptomycin. Erythrocytes were removed by incubating with red blood cell lysis buffer (Sigma Aldrich). Cellular content was assessed by cell counting using a Cellometer Auto T4 Cell Counter (Nexcelom Bioscience) in combination with multicolor flow cytometry.

### Flow cytometry

Equal numbers of cells or 20 μL of blood was stained for each sample. Blood samples were mixed and washed with Hank’s buffered saline solution containing 2 mM EDTA (Life Technologies). Cells were stained with LIVE/DEAD cell viability assay (Life Technologies). All samples were then blocked with 5 μg/mL anti- CD16/32 (2.4G2; produced in-house) and heat-inactivated normal mouse serum (1:10) in FACS buffer (0.5% BSA and 2 mM EDTA in Dulbecco’s PBS) before surface staining on ice with antibodies to F4/80 (BM8), Siglec-F (E50-2440), Ly-6C (HK1.4), Ly-6G (1A8), Gr-1 (RB6-8C5), B220 (RA3-6B2), TCRβ (H57-597), CD11b (M1/70), CD11c (N418), I-A/I-E (M5/114.15.2), CD19 (eBio1D3 or 6D5), CD4 (GK1.5), CD8α (53–6.7), CD115 (AFS98), CD45.1 (A20), or CD45.2 (104; eBioscience or BD). Erythrocytes in blood samples were lysed using FACS Lyse solution (BD Biosciences). All antibodies were purchased from Biolegend UK unless stated otherwise.

Detection of intracellular Relm-α and NOS2 was performed directly *ex vivo*. Cells were stained for surface markers then fixed with 2% paraformaldehyde (Sigma Aldrich) and permeabilized using Permeabilization Buffer (eBioscience). Cells were then stained with purified polyclonal rabbit anti-Relm-α (PeproTech) or directly labeled Abs to NOS2 (CXNFT; eBioscience), followed by Zenon anti–rabbit reagent (Life Technologies). Expression of Relm-α and NOS2 was determined relative to appropriate polyclonal or monoclonal isotype control.

Samples were acquired on a BD LSR II or BD FACSCanto II using BD FACSDiva software (BD Bioscience) and post-acquisition analysis performed using FlowJo v9 software (Tree Star Inc.).

### Partially protected bone marrow chimeras

Bone marrow chimeric mice were constructed by exposing anaesthetized C57BL/6 Cd45.1 mice to a single dose of 11.5 cGy γ radiation while shielding the abdomen, thorax, head and fore limbs with a 2-inch lead screen followed by i.v. injection of 5 x 10^6 donor bone marrow cells from congeneic Cd45.2 mice. Chimeric mice were left for 8 weeks before further experimental manipulation.

### Cell-culture experiments

For in vitro conversion experiments *H*. *polygyrus*-, SL3261- or thioglycollate-elicited PEC were counted as described above and seeded to 96-well plates at 2x10^5 cells per well in RPMI 1640 containing 5% foetal bovine serum, 2 mM L-glutamine, 100 U/mL penicillin and 100 μg/mL streptomycin and stimulated with murine recombinant IL-4 (rIL-4, 20ng/mL, Peprotech), lipopolysaccharide (LPS, 100ng/mL; *Escherichia coli* 0111:B4; Sigma-Aldrich) or medium alone for 24h and analysed for MΦ activation markers by flow cytometry.

### RNA-isolation and microarray analysis

MΦ were purified using FACS-sorting on a FACSAria cell sorter (BD Biosciences) according to their expression of surface molecules (F4/80+, SiglecF-, CD11b+, CD11c-, B220-, CD3-; all antibodies purchased from BioLegend or eBioscience) reaching purities of above 96%. Isolated MΦ were frozen at -70C and total RNA isolated using RNeasy mini columns (Qiagen). Sample preparation, quality control, running the microarray and initial bioinformatics analysis were carried out by the Bioinformatics and Genomic Technologies Core Facilities at the University of Manchester. In brief 10 ng of total RNA were converted to cDNA using the GeneChip WT Pico Kit (Affymetrix) and hybridized to Affymetrix GeneChip Mouse Gene 1.0 ST Array according to the manufacturer’s instructions. Mouse Transcriptome Assay 1.0 data were processed and analysed using Partek Genomics Solution (version 6.6, Copyright 2009, Partek Inc., St. Charles, MO, USA) with the following options: probesets were quantile normalised and RMA background correction applied. Probesets were summarised to genes by calculating the means (log 2). Validation and gene enrichment strategies consisted of the following steps. Step 1, to establish relationships and compare variability between replicate arrays and experimental conditions, principal components analysis (PCA) was used. PCA was chosen for its ability to reduce the effective dimensionality of complex gene-expression space without significant loss of information [54]. Step 2, Differential expression analysis was performed on annotated genes with Limma using the functions lmFit and eBayes [55]. Gene lists of differentially expressed genes were controlled for false discovery rate (fdr) errors using the Benjamini–Hochberg procedure [56]. Step 3, functional annotation of gene lists containing significantly differentially expressed genes was done with QIAGEN’s Ingenuity Pathway Analysis (IPA, QIAGEN Redwood City, www.qiagen.com/ingenuity). Microarray data have been deposited to NCBI's Gene Expression Omnibus and are accessible through GEO Series accession number GSE85805.

### Statistical analysis

Statistical analysis was performed using Prism 6 for Mac OS X (v6.0f, GraphPad Software). Differences between groups were determined by t-test or ANOVA followed by Tukey’s or Dunn’s multiple comparison-test. In some cases data was log-transformed to achieve normal distribution as determined by optical examination of residuals. Where this was not possible a Mann-Whitney or Kruskal-Wallis test was used. Comparison of activation markers of F4/80^high / low^ or Relm-α positive / negative MΦ within one experimental animal were considered as paired observations. Differences were assumed statistically significant for *P* values of less than 0.05.

## Supporting information

S1 Fig (A & B) Repeat experiments of Fig 1A. 5 mice per group. (C) Transfer of M(IL-4) (from IL-4 complex injected donors) into SL3261 infected animals and analysed 24 h later by flow cytometry. 1 experiment of 1. 5 animals per group.
(TIF)

S2 Fig Gating strategy to identify peritoneal immune cells in consecutive co-infection experiments. Lineage: CD19^+^, TCRβ^+^, Ly6G^+^, SiglecF^+^.
(TIF)

S3 Fig (A) Schematic depiction of the consecutive co-infection model utilised in this study. (B) Number of peritoneal MΦ in *H*.*polygyrus* infected mice nine and 14 days after infection.
(TIF)

S4 Fig Previous *H*. *polygyrus* co-infection does not alter resistance to high dose SL3261 infection. 1 experiment of 1. 5 animals per group.
(TIF)

S5 Fig Schematic depiction of partially protected chimera generation and infection. (TIF)

S6 Fig Percent Annexin V + of F4/80^high^ (black bars) or F4/80^low^ MΦ (grey bars) in single or coinfected C57BL/6 animals. Bars represent mean and SEM of 5 mice per group. One representative experiment of 2 shown.
(TIF)

S7 Fig Hierarchical clustering of global gene expression in MΦ from single or co-infected C57BL/6 mice. (TIF)

S8 Fig Venn diagram depicting the number of overlapping differentially expressed genes in F4/80^low^ and F4/80^high^ MΦ from naïve (A) and co-infected mice (B). (TIF)

S9 Fig Selected pathways enriched with differentially expressed genes in F4/80^high^ MΦ from co-infected animals as compared to F4/80^high^ MΦ from single *H*. *polygyrus* infected animals. (TIF)

S1 Table List of differentially expressed genes between F4/80^high^ and F4/80^low^ MΦ unique to naive or co-infected animals or differentially expressed under both conditions. (XLS)
