# Supplementary figures and images for: Macrophage origin limits functional plasticity in helminth-bacterial co-infection
Source: PLoS Pathog. 2017 Mar 23;13(3):e1006233. doi: 10.1371/journal.ppat.1006233 (PMC5364000; doi:10.1371/journal.ppat.1006233)

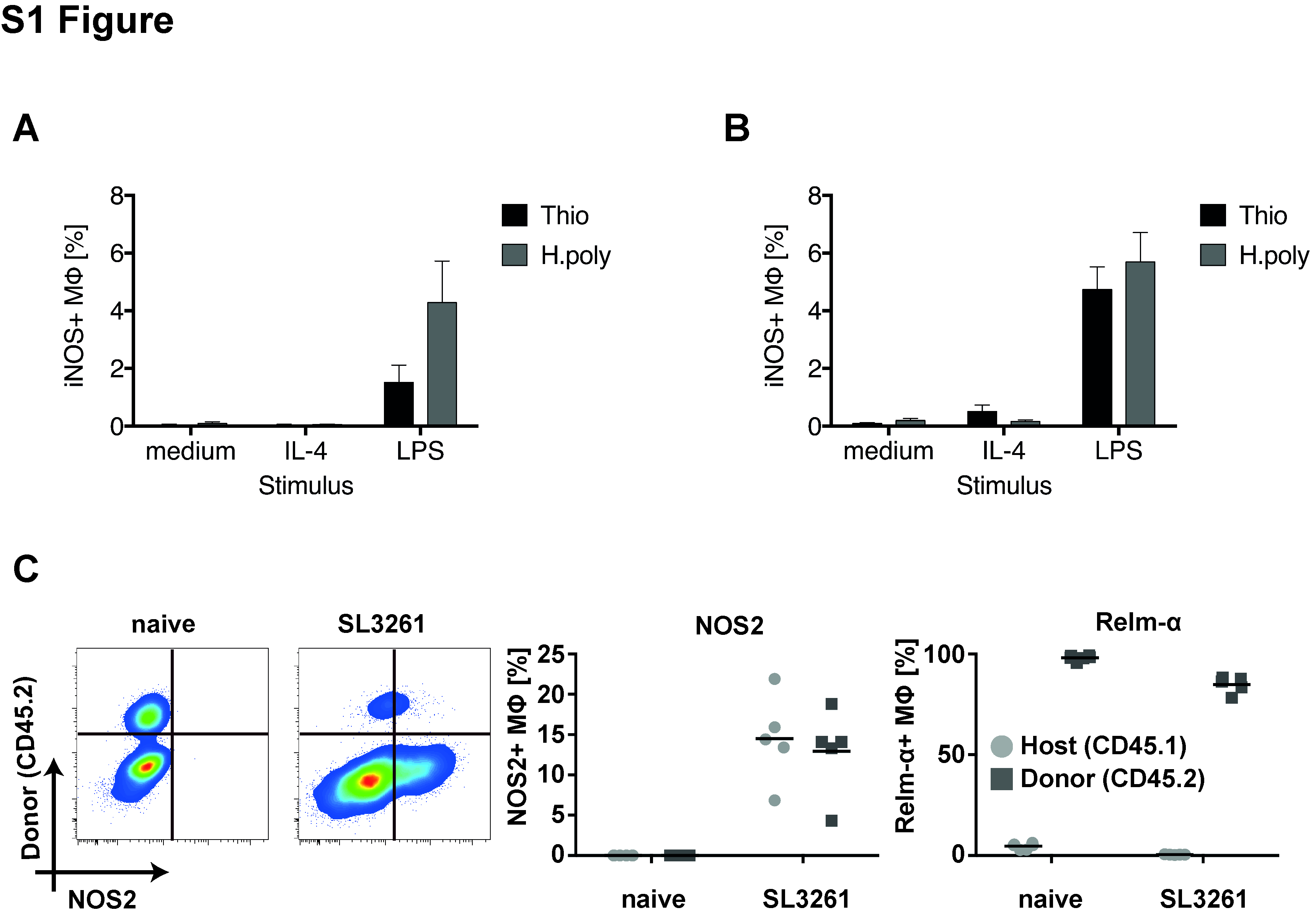

Supplement: S1 Fig — (A & B) Repeat experiments of Fig 1A. 5 mice per group. (C) Transfer of M(IL-4) (from IL-4 complex injected donors) into SL3261 infected animals and analysed 24 h later by flow cytometry. 1 experiment of 1. 5 animals per group. (TIF) [file ppat.1006233.s001.tif]

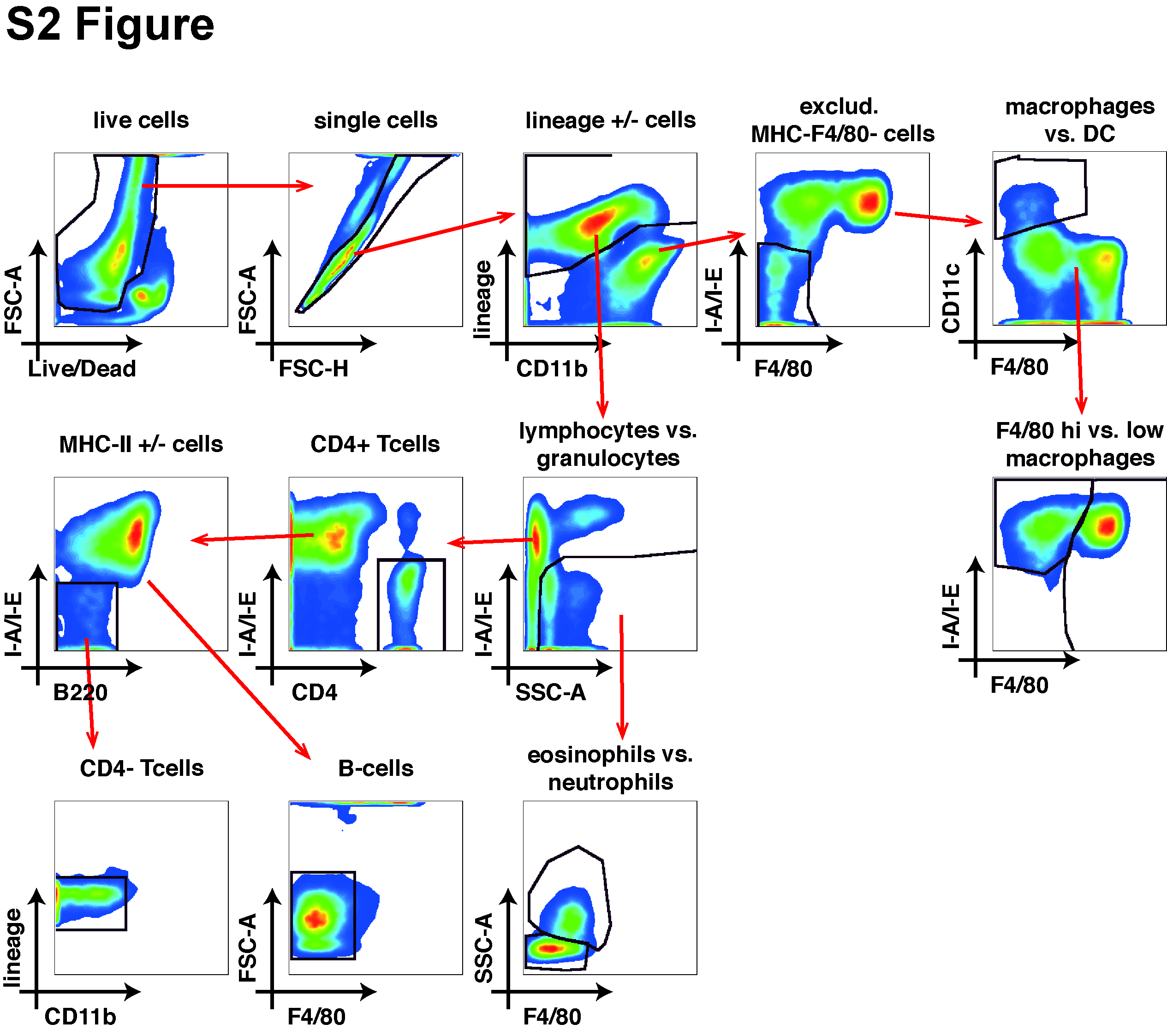

Supplement: S2 Fig — Lineage: CD19+, TCRβ+, Ly6G+, SiglecF+. (TIF) [file ppat.1006233.s002.tif]

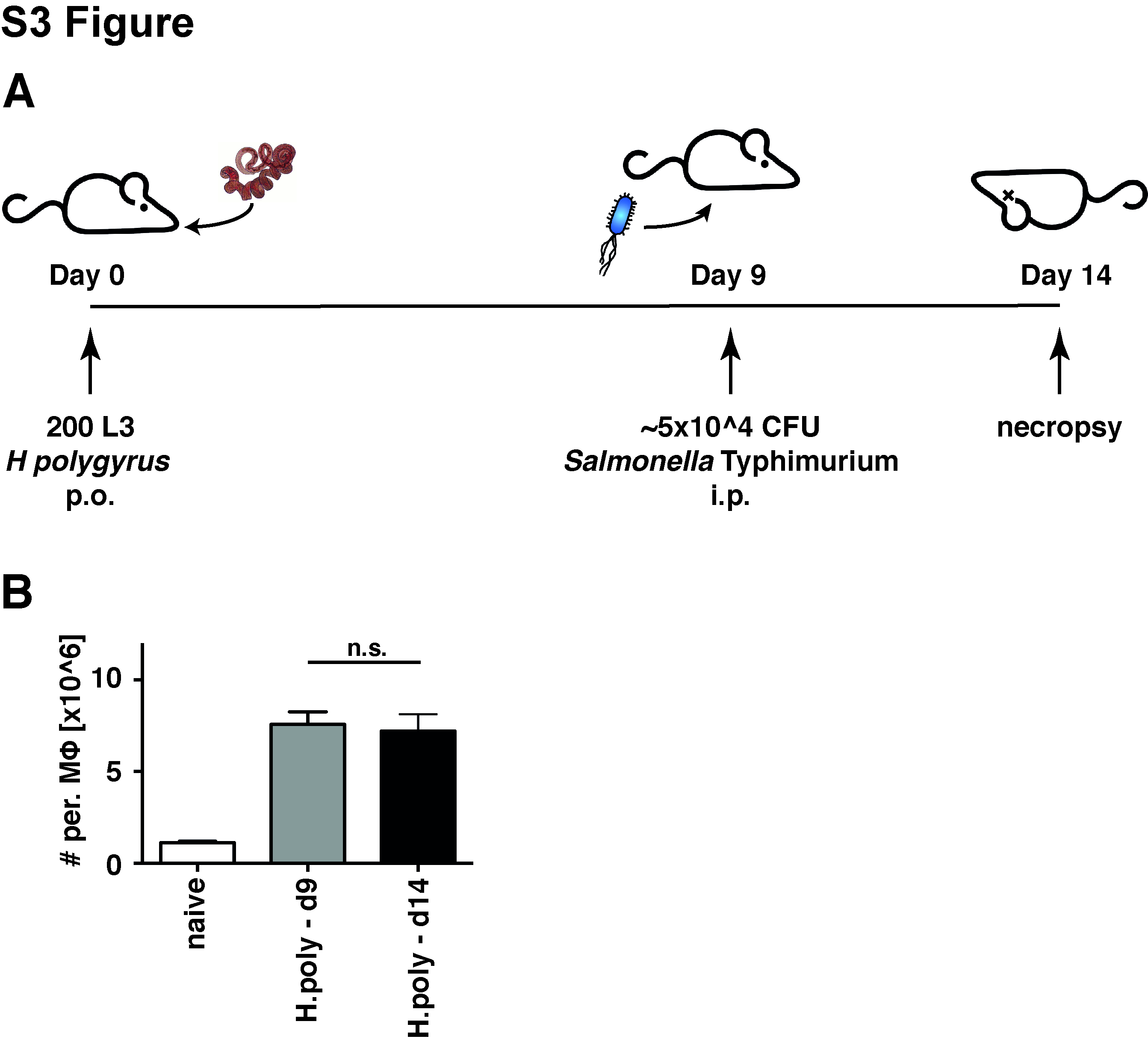

Supplement: S3 Fig — (A) Schematic depiction of the consecutive co-infection model utilised in this study. (B) Number of peritoneal MΦ in H.polygyrus infected mice nine and 14 days after infection. (TIF) [file ppat.1006233.s003.tif]

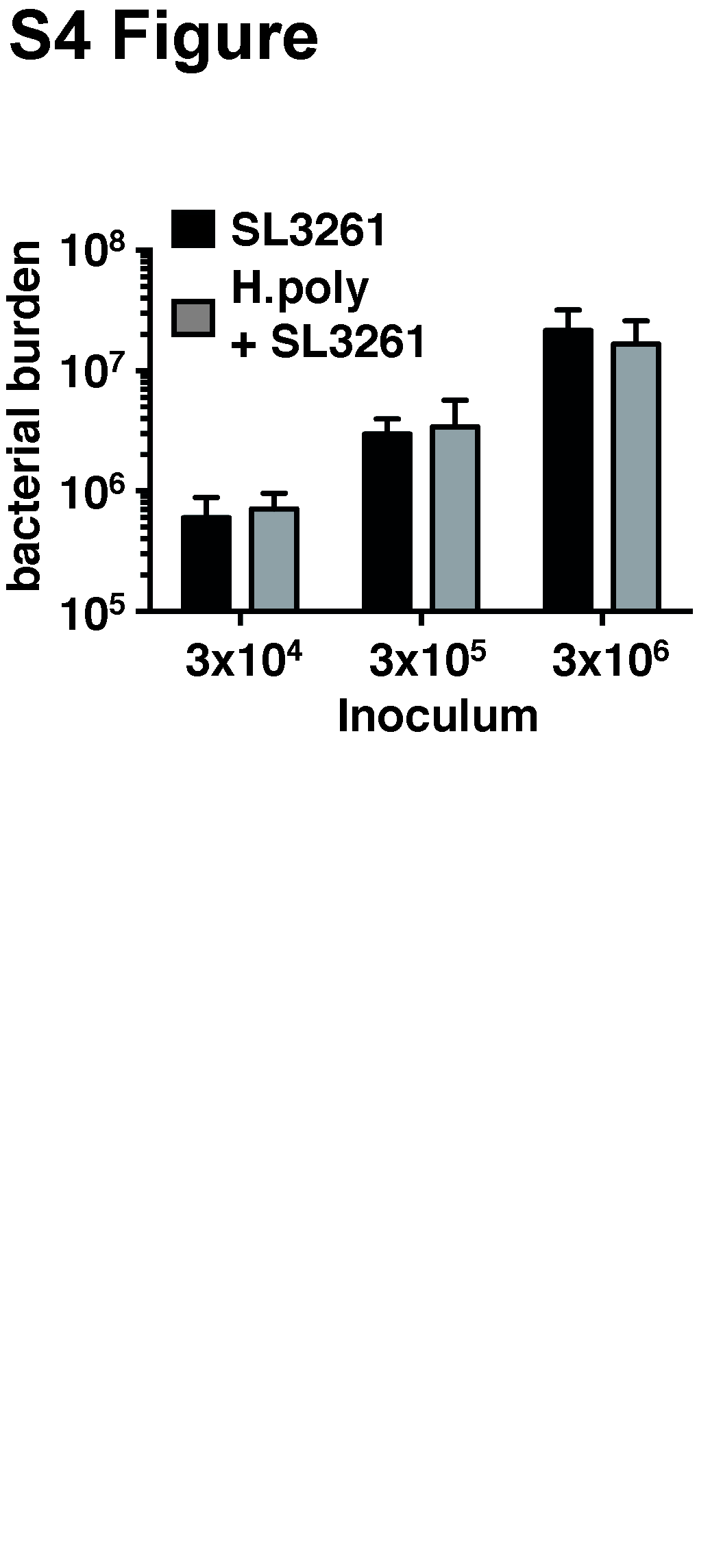

Supplement: S4 Fig — 1 experiment of 1. 5 animals per group. (TIF) [file ppat.1006233.s004.tif]

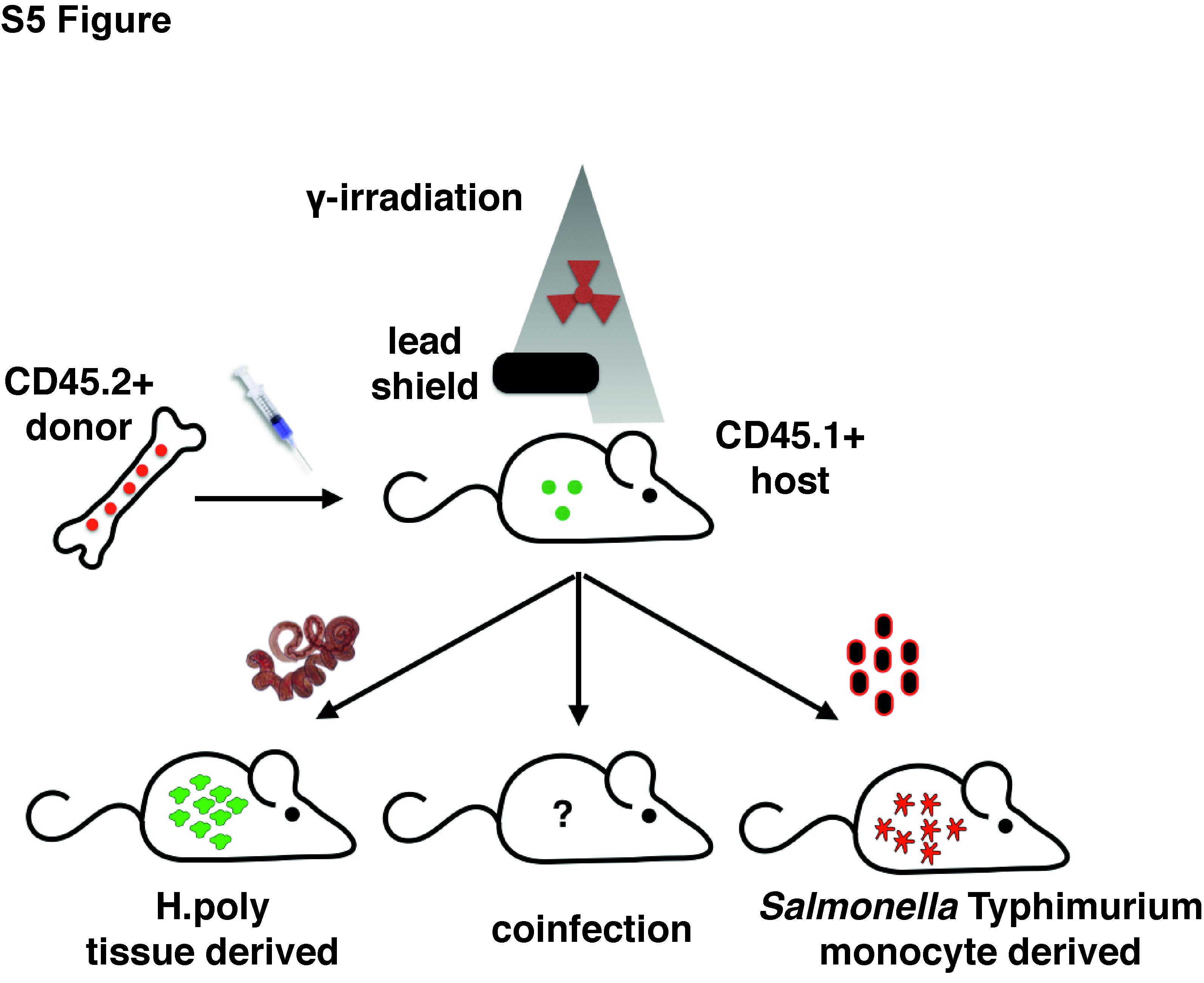

Supplement: S5 Fig — (TIF) [file ppat.1006233.s005.tif]

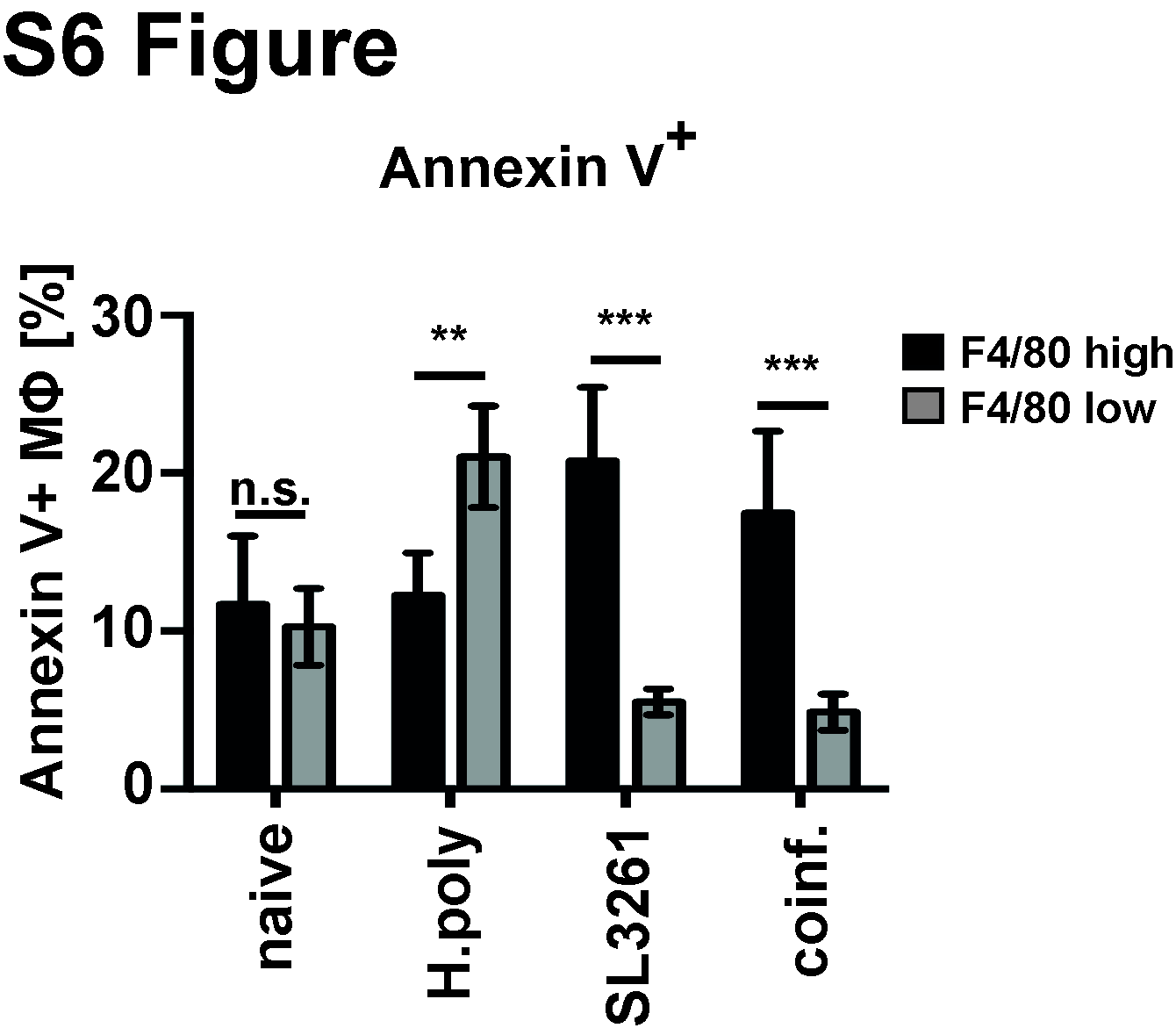

Supplement: S6 Fig — Bars represent mean and SEM of 5 mice per group. One representative experiment of 2 shown. (TIF) [file ppat.1006233.s006.tif]

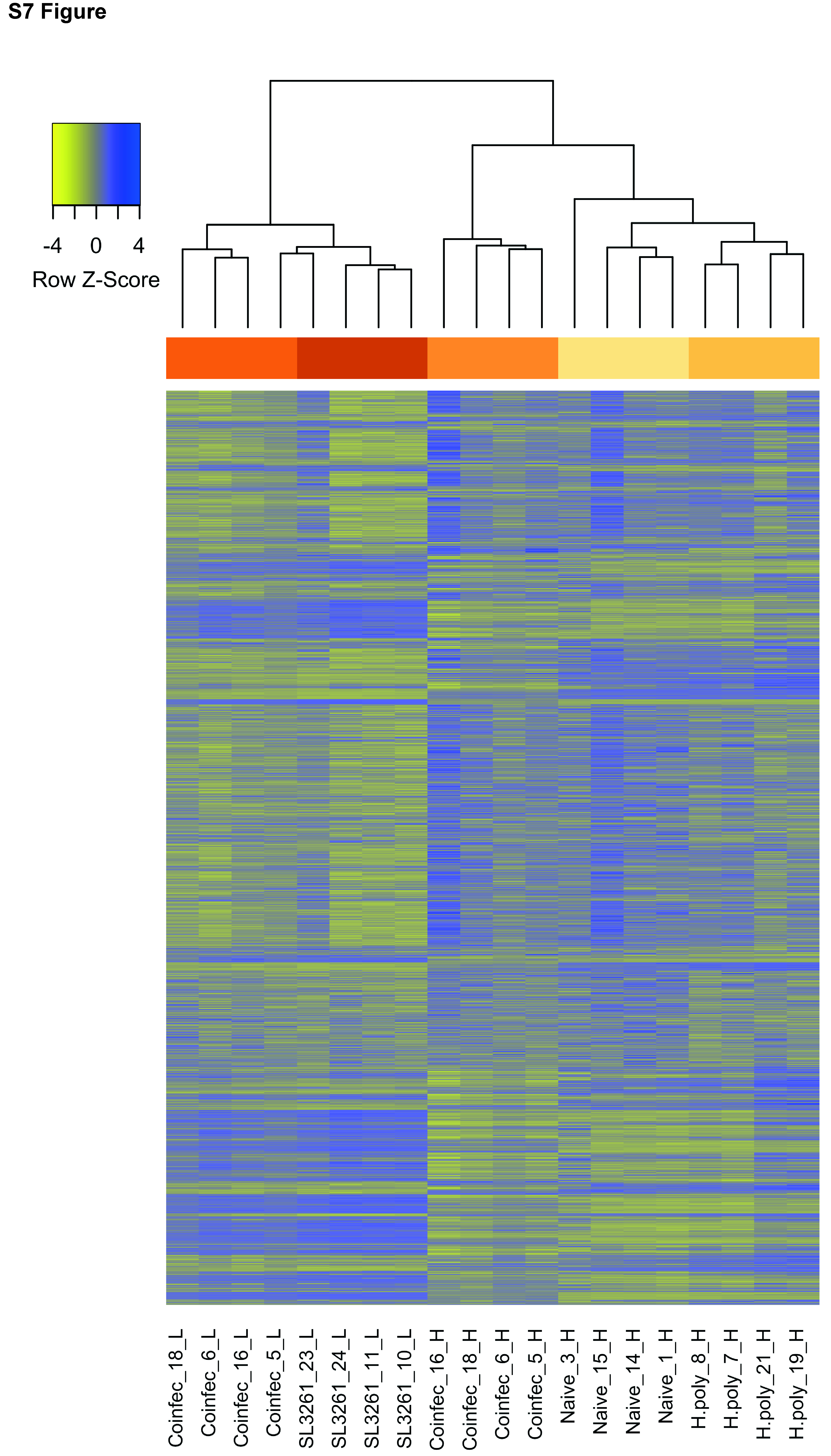

Supplement: S7 Fig — (TIF) [file ppat.1006233.s007.tif]

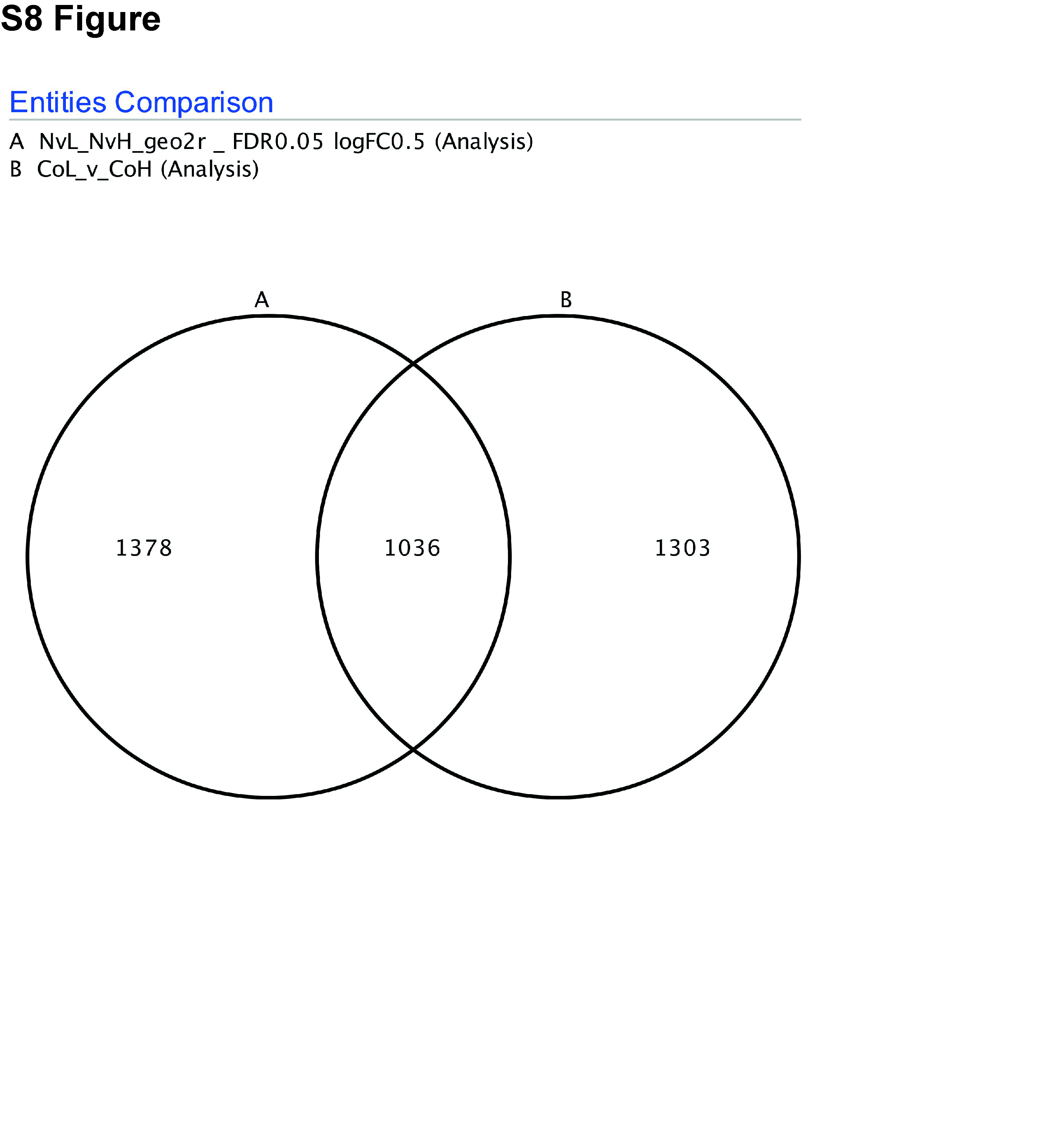

Supplement: S8 Fig — (TIF) [file ppat.1006233.s008.tif]

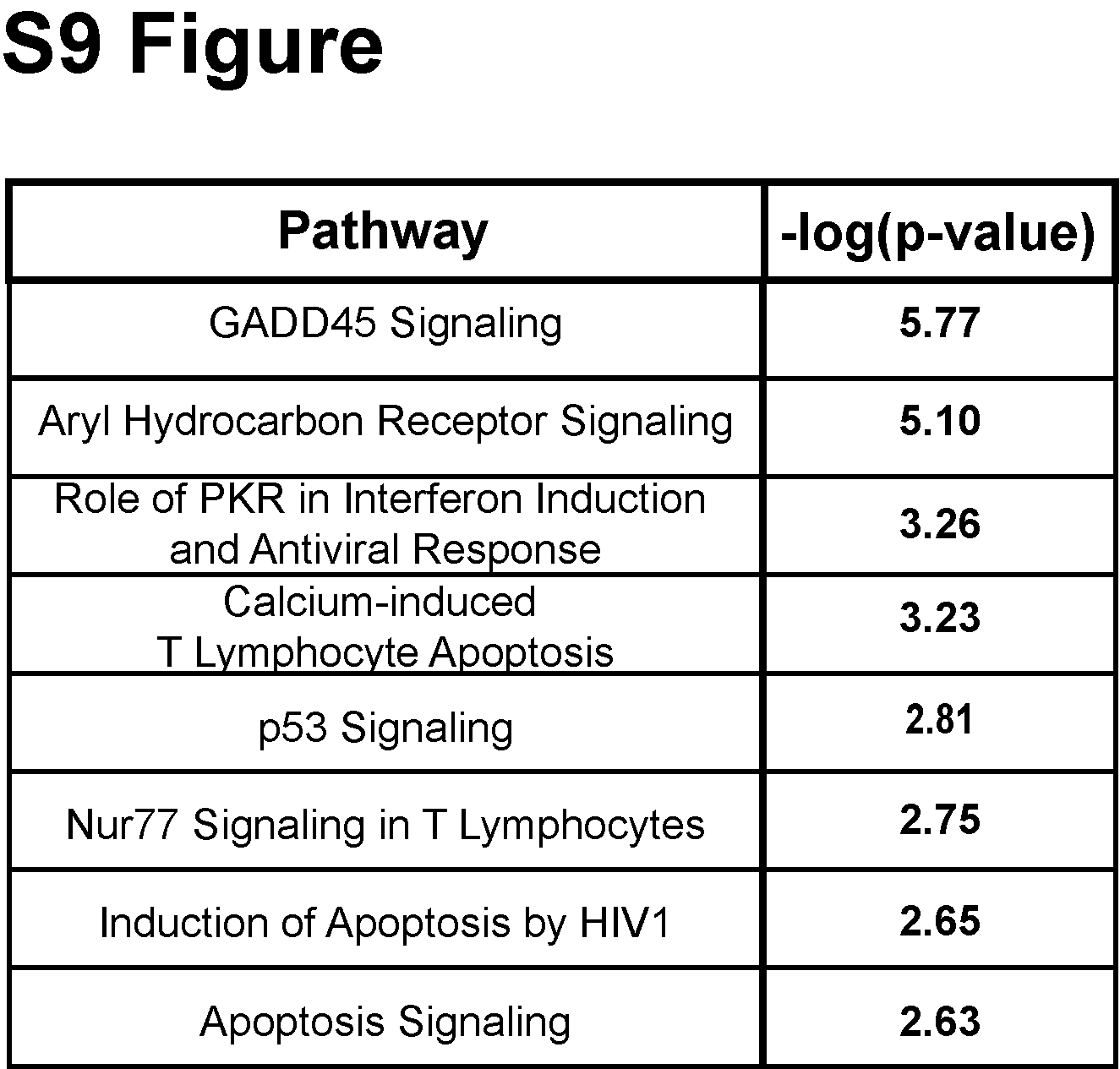

Supplement: S9 Fig — (TIF) [file ppat.1006233.s009.tif]
